# Electrical and Optical Properties of γ-SnSe: A New Ultra-narrow Band Gap Material

**DOI:** 10.1021/acsami.2c22134

**Published:** 2023-03-15

**Authors:** Noy Zakay, Adi Schlesinger, Uri Argaman, Long Nguyen, Nitzan Maman, Bar Koren, Meital Ozeri, Guy Makov, Yuval Golan, Doron Azulay

**Affiliations:** † Department of Materials Engineering, Ben-Gurion University of the Negev, Beer-Sheva 8410501, Israel; ‡ Ilse Katz Institute for Nanoscale Science and Technology, Ben-Gurion University of the Negev, Beer-Sheva 8410501, Israel; § Azrieli College of Engineering, Jerusalem 9103501, Israel; ∥ Racah Institute of Physics, The Hebrew University, Jerusalem 9190401, Israel

**Keywords:** SnSe, thin films, electrical properties, ultranarrow band gap, solution deposition

## Abstract

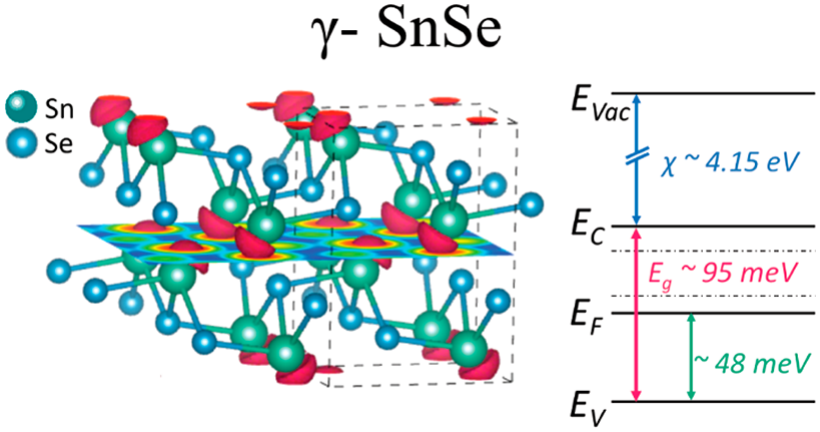

We describe the unusual properties of γ-SnSe, a new orthorhombic binary phase in the tin monoselenide system. This phase exhibits an ultranarrow band gap under standard pressure and temperature conditions, leading to high conductivity under ambient conditions. Density functional calculations identified the similarity and difference between the new γ-SnSe phase and the conventional α-SnSe based on the electron localization function. Very good agreement was obtained for the band gap width between the band structure calculations and the experiment, and insight provided for the mechanism of reduction in the band gap. The unique properties of this material may render it useful for applications such as thermal imaging devices and solar cells.

## Introduction

Tin monoselenide (SnSe) is an attractive semiconductor for applications in photovoltaic and optoelectronic devices^1−8^ as well as for thermoelectric applications.^9−23^ The stable polymorph under standard temperature and pressure conditions is the α-SnSe phase that has an indirect band gap of 0.9–1.0 eV.^2,3,24^ It has a high absorption coefficient of ∼10^5^ cm^–1^ and high hole mobility^24^ that makes it suitable for use as an absorber in solar cells. Its low thermal conductivity and high electrical conductivity make it an excellent thermoelectric material with a figure of merit, defined as ZT = *S*^2^σ*T*/κ, as high as ∼2.6 at 923 K,^11,25^ where *S* is the Seebeck coefficient, σ is the electrical conductivity, *T* is the absolute temperature, and κ is the thermal conductivity.^26,27^ It has been shown that under high pressure there is a reduction of the energy gap of the material and improvement in its electrical conductivity up to a phase transition from semiconductor to semimetal at ∼10 GPa.^28,29^ Such an improvement in electrical conductivity improves the ZT of the thermoelectric device. Another phase of recent interest is the metastable cubic phase, π-SnSe, with a large unit cell *a*_0_ of 1.1970 nm and an indirect band gap of 1.28 eV.^30,31^ Recently, we reported on solution deposition of thin films of an additional new and previously unreported orthorhombic metastable phase, γ-SnSe, with the following lattice constants: *a*_0_ = 0.8332 nm, *b*_0_ = 0.4136 nm, and *c*_0_ = 0.6115 nm.^32^

While the electrical and optical properties of α-SnSe have been widely investigated,^33−42^ the properties of γ-SnSe have yet to be studied. In this paper, we report, for the first time, on the properties of the γ phase, which is an ultranarrow band gap, highly conductive material under standard pressure and temperature conditions. These properties make it suitable for various applications, including infrared (IR) detectors, thermal imaging devices, and thermoelectric devices.^43^ In this study, we used a combination of structural, optical, and electrical characterization techniques to study the properties of γ-SnSe. The experimental measurements were found to be in good agreement with calculations of the band structure of the material. The projected density of states (DOS) and electron localization function (ELF) using density functional theory (DFT) provided additional insight into the structure and bonding in this phase.

## Experimental Details

### Materials

Pb(NO_3_)_2_ (≥99.0%), SnCl_2_·2H_2_O (≥99.99%), thiourea (≥99.0%), sodium hydroxide (≥98%), triethanolamine (≥99.0%), sodium sulfite (≥98%), and selenium powder (≥99.99%) were purchased from Sigma-Aldrich and used without further purification. Acetone (technical grade), glacial acetic acid (A.R.), and isopropanol (99.8%) were purchased from Bio-Lab and used without further purification. Distilled water (DIW) was obtained using a Millipore Direct Q3 water purification system. Monocrystalline GaAs (100) wafers (epi-polished, ±0.1° miscut, undoped) were manufactured by AXT Inc. and purchased from Geo Semiconductor (UK) Ltd. (Fremont, CA). Amorphous quartz wafers were purchased from Ted Pella Inc. A stock solution of sodium selenosulfate (Na_2_SeSO_3_, 0.2 M) was prepared with sodium sulfite (0.5 M) mixed with selenium powder in DIW and stirred at 90 °C for 3 h. This solution was filtered to remove unreacted selenium powder and used for no longer than 5 days.

### Substrate Preparation

Double-side-polished GaAs (100) wafers and amorphous quartz wafer substrates were cleaved into 2 cm × 2 cm rectangles and sonicated for 10 min at 40 °C in a Contrad 70 detergent solution (Decon Laboratories). The substrates were rinsed with DIW, acetone, and isopropanol and then dried under a N_2_ flow.

### Substrate Pretreatment

Beakers were prepared for carrying out substrate surface pretreatments with 30 mM Pb(NO_3_)_2_ and 1.2 M NaOH in a final volume of 50 mL. The pretreatments were carried out at 30 °C for 10 min.^32,44^

The role of this pretreatment is to deposit an ultrathin layer of adsorbed Pb^2+^ ions on the substrate surface to enhance wetting of the films and their uniformity. The thickness of the Pb^2+^ layer present on the surface was reported to be <2 Å.^44^ Note that the substrate pretreatment was carried out without adding a sulfide precursor source to prevent the formation of a PbS film.

### Deposition Procedure

After the pretreatment, the substrate was rinsed with water and immediately transferred into the SnSe deposition bath. The detailed protocols for solution deposition of γ-SnSe thin films were described by Koren et al.^32^

The stock solution contained 0.35 g of SnCl_2_·2H_2_O in a solution containing 3.5 mL of DIW and 1.5 mL of glacial acetic acid. This solution inserts into the deposition beaker with final concentrations of 45 mM SnCl_2_·2H_2_O, 0.61 M glacial acetic acid, 1.55 M triethanolamine (TEA), and 1.16 M NaOH. The substrates were immersed in the deposition solution immediately after initiating the reaction by adding Na_2_SeSO_3_ to reach a final concentration of 9 mM. The final bath volume was 40.3 mL.

Two different substrates were used in this work. Quartz, an insulating substrate, is appropriate for electrical characterization, while GaAs is well-suited for optical characterization in the infrared range. Depositions were carried out at 0 °C for 24 h for the GaAs substrates; four successive depositions were required to reach the desired thickness. For amorphous quartz wafers, depositions were carried out at 20 °C for 1.5 h. Also here, four successive depositions were required. The different temperature and deposition time conditions used for the different substrates were selected after optimization and showed the best results.

### Characterization

#### X-ray Diffraction (XRD)

XRD characterization was carried out using a Panalytical Empyrean diffractometer using Cu Kα radiation (λ = 1.5405 Å) equipped with a position-sensitive X’Celerator detector. Data were collected at 40 kV and 30 mA. Diffraction patterns were taken in a 2θ range of 20–60° in 6.25°/min steps. For GaAs substrates, the measurements were conducted with a sample offset angle of 2° to prevent masking of the signal from the films by the monocrystalline substrate.

#### High-Resolution Scanning Electron Microscopy (HRSEM)

Images were obtained using an FEI Verios 460L HRSEM instrument in plan view and cross section sample geometries. Acceleration voltages ranged from 3 to 5 kV, and beam currents of 25–50 pA were used.

#### Dual-Beam FIB

The film thickness was measured using a dual-beam FIB/SEM tool (Thermo-Fisher Helios G4 UC). First, the regions of interest (ROI) were coated with two different layers: 500 nm electron deposition of Pt to protect the ROI from the ion beam and 2 μm ion deposition of carbon for protection and to enable straight cut and cleaning of the cross section without curtaining effects. Subsequently, we used the ion beam (30 kV Ga) to mill near the protected area until the film/substrate interfaces appeared in the SEM. After milling, the cross-section images were cleaned with gradually decreasing probe currents until good quality imaging is achieved, from which the film thickness was evaluated.

#### Optical Measurements

Transmission IR measurements were performed using a Bruker VERTEX 80V Fourier transform infrared (FTIR) spectrometer. The scans used a room-temperature deuterated triglycine sulfate detector ranging from 1 to 16 μm.

#### DFT Calculations

Calculations were performed using the Quantum Espresso simulation package^45^ within the DFT framework with ultrasoft pseudopotentials. The pseudopotentials are taken from the Garrity, Bennett, Rabe, and Vanderbilt (GBRV) high-throughput library.^46^ The exchange correlation is approximated by the Perdew–Burke–Ernzerhof (PBE) functional.^47^ Plane-wave expansion was cut off at 40 Ry; the cutoff for density and potential was 200 Ry, and k-point sampling was performed on an 8 × 8 × 8 grid. With these numerical parameters, the total energy converges to 10^–6^ Ry/atom, and the band gap converges to 0.03 eV. In the variable-cell relaxation process, the stress converges up to 0.5 kbar and the forces converge up to 10^–3^ a.u. The Heyd–Scuseria–Ernzerhof (HSE)^48,49^ calculation employed the same pseudopotentials and atomic geometry as the PBE calculation, k-point meshes of 6 × 6 × 6 and 1 × 2 × 2 for the exact exchange calculation, and values of 60 and 240 Ry for the cutoff for wave functions and density and potential, respectively.

#### Electrical Conductivity and Hall Measurements

Electrical conductivity and Hall effect measurements were performed using a ^4^He cryostat. The film conductivity was measured using a four-probe configuration. Hall measurements were carried out using a Hall bar configuration with a magnetic field of ±2T for four different currents: 15 nA, 0.5 μA, 1 μA, and 3 μA. For both measurements, Au contacts were deposited on top of the films. A schematic illustration of the Hall measurements setup is presented in Figure S4.

## Results and Discussion

**Figure 1:**
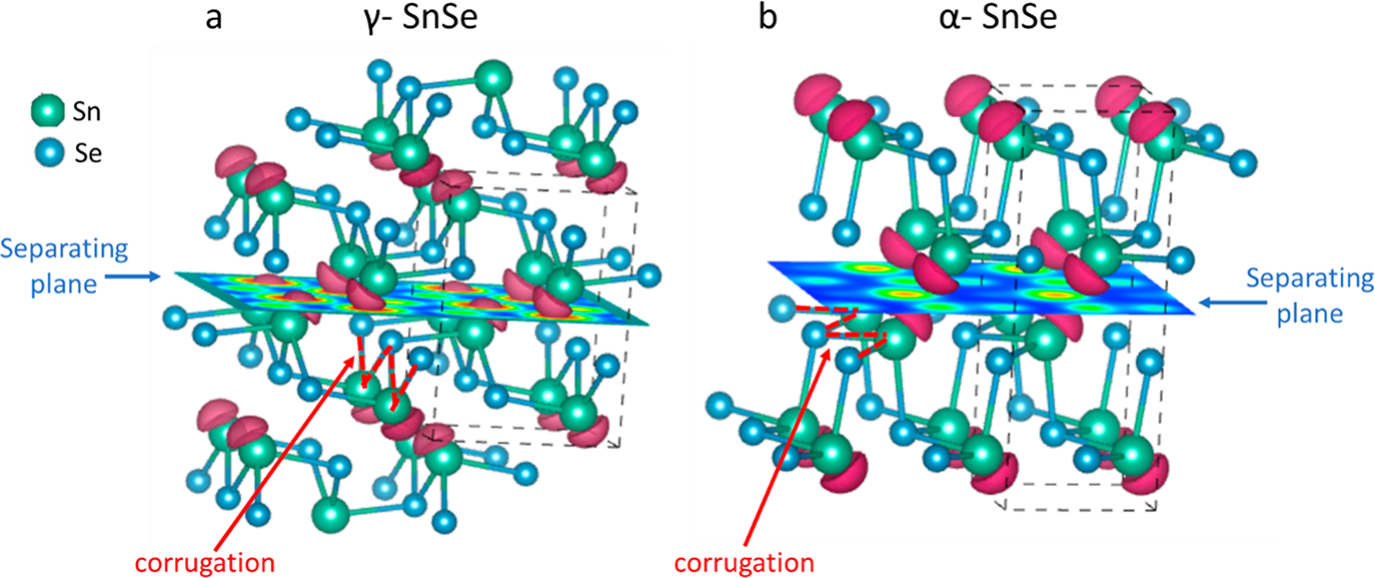
Illustration of (a) the γ-SnSe structure and (b) the α-SnSe structure. The dashed black lines outline the unit cells. The dashed red lines indicate the zigzag corrugation. The colored plane shows the electron localization function (ELF) on the separating plane. The red hemispheres are 0.92 ELF isosurfaces.

The γ phase is an orthorhombic structure like the α phase.^32,33^ These phases are both layered structures with a corrugation (“zigzag”) pattern. However, the difference between these phases lies in the orientation of the corrugation relative to the plane that separates the layers. Figure 1a shows that the corrugation is perpendicular to the plane that separates the layers in the γ phase. In contrast, the corrugation in the α phase is parallel to this plane (Figure 1b). To analyze the electron distribution that characterizes the two phases, we calculated the electron localization function (ELF), also shown in Figure 1. The results indicate that the lone pairs of the tin atoms are located between the layers in both phases, albeit closer in the γ phase. The geometry of the γ phase, i.e., the orientation of the zigzag chain in the layers, allows the γ phase layers, 4.28 Å thick, to be thinner than the layers in the α phase, which are 5.92 Å thick. It also offsets the position of the lone pairs of adjacent layers on the separating plane, allowing them to be closer in the γ phase.

**Figure 2:**
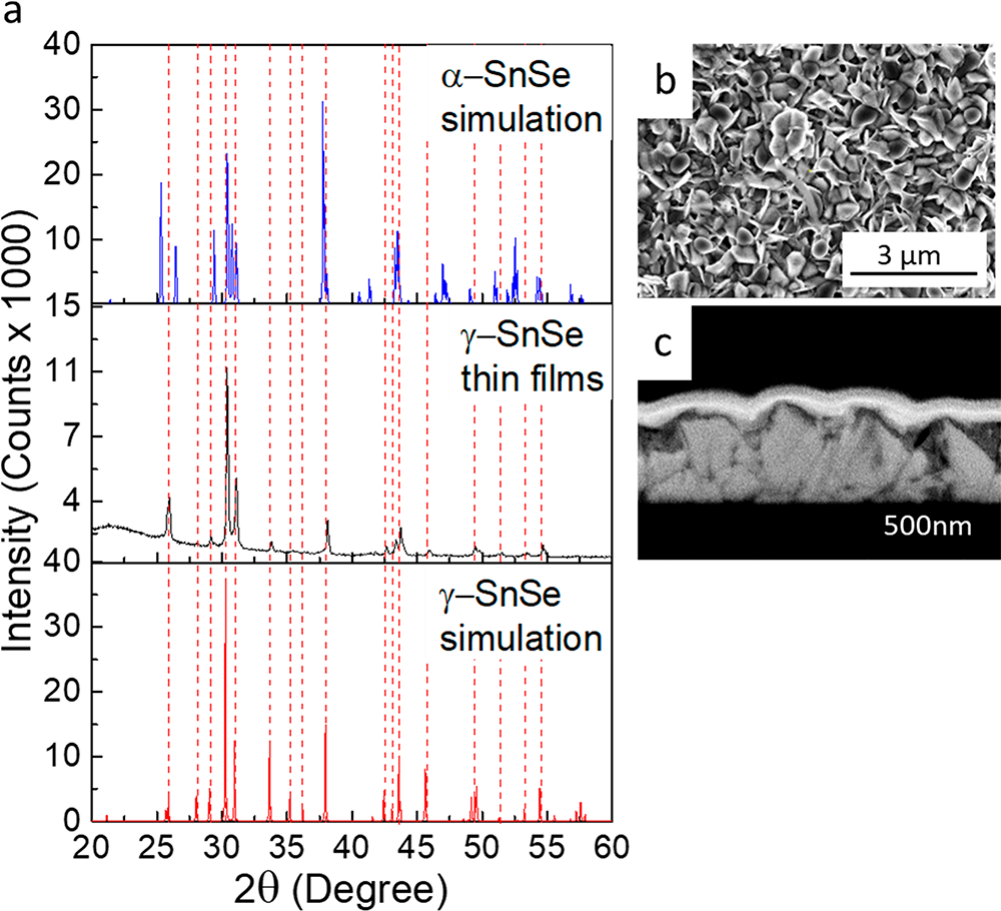
Characterization of γ-SnSe thin films deposited onto quartz substrates. (a) X-ray diffractogram of simulated diffraction pattern of α-SnSe, experimental γ-SnSe thin films, and simulated diffraction pattern of γ-SnSe. Corresponding HRSEM images of γ-SnSe deposited onto quartz in (b) plan view and (c) cross section. Dashed hairlines in red correspond to the orthorhombic γ-SnSe phase. Top and bottom plates in panel a (simulation of the α and γ phases) were reproduced with permission from ref (32). Copyright 2021 Royal Society of Chemistry.

To study the electrical properties of the films, we deposited γ-SnSe thin films on quartz substrates. These substrates, however, cannot be used for optical absorbance measurements due to the absorption of quartz in the studied wavelength range (3–13 μm). Therefore, we used intrinsic GaAs (100) substrates, transparent in this range for the optical measurements. XRD patterns of SnSe films deposited on these two substrates indicate that in both cases, the film structure is γ-SnSe, as shown in Figure 2a and Figure S1a. Panels b and c of Figure 2 present HRSEM images of a γ-SnSe film deposited on a quartz substrate (in plan view and cross section, respectively). In addition, panels b and c of Figure S1 show HRSEM images for γ-SnSe films on GaAs. The results show that for both substrates, the films are continuous, compact, and polycrystalline and have no apparent preferred orientation. There are differences in the typical grain size, i.e., approximately 150–250 nm for the GaAs substrate and 250–300 nm for the quartz substrate, and in film thickness (∼650 nm on quartz and ∼550 nm on GaAs). In addition, atomic force microscopy (AFM) topography images (Figure S2) support these results and show agreement with the HRSEM images.

**Figure 3:**
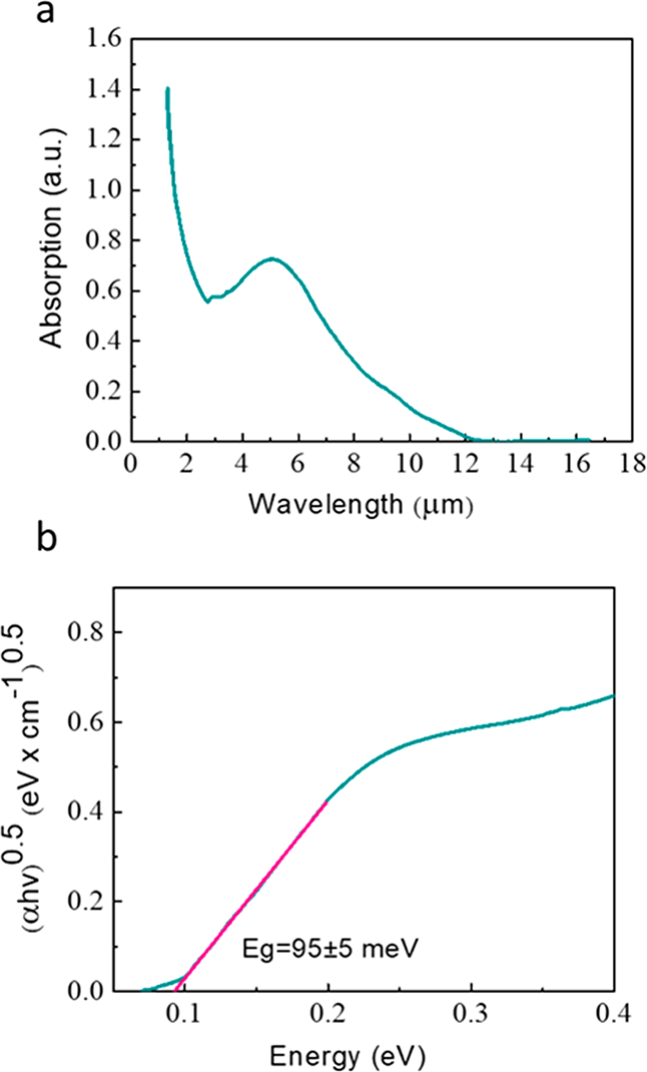
(a) Absorption measurements were performed on γ-SnSe thin films deposited onto the GaAs substrate. (b) Tauc plots were constructed to extract the band gap for each sample.

To evaluate the energy gap of this new phase, we measured the optical absorbance of γ-SnSe deposited on the GaAs substrate. The results, presented in Figure 3a, show an absorption onset at ∼12.4 μm. Figure 3b displays a Tauc plot corresponding to an indirect band gap, in agreement with the DFT calculations reported below. We note that the influence of the substrate has been subtracted from the spectrum shown in Figure 3a. Moreover, comparison with the absorption spectrum of an uncoated GaAs substrate reported in previous work from our group rules out the possibility that the features assigned to γ-SnSe arise from the substrate.^50^ In addition, panels a and b of Figure S3 present the absorption spectrum in a logarithmic scale and the absorption coefficient as a function of wavelength, respectively.

The estimated band gap of this phase is ∼95 ± 5 meV, much smaller than that reported for other SnSe phases, 0.9–1.0 eV for the α phase and 1.28 eV for the π phase.^30,33,39,40^ While differences in energy gaps are expected between phases, such a dramatic change is surprising and can lead to novel properties and, consequently, applications of this material.

**Figure 4:**
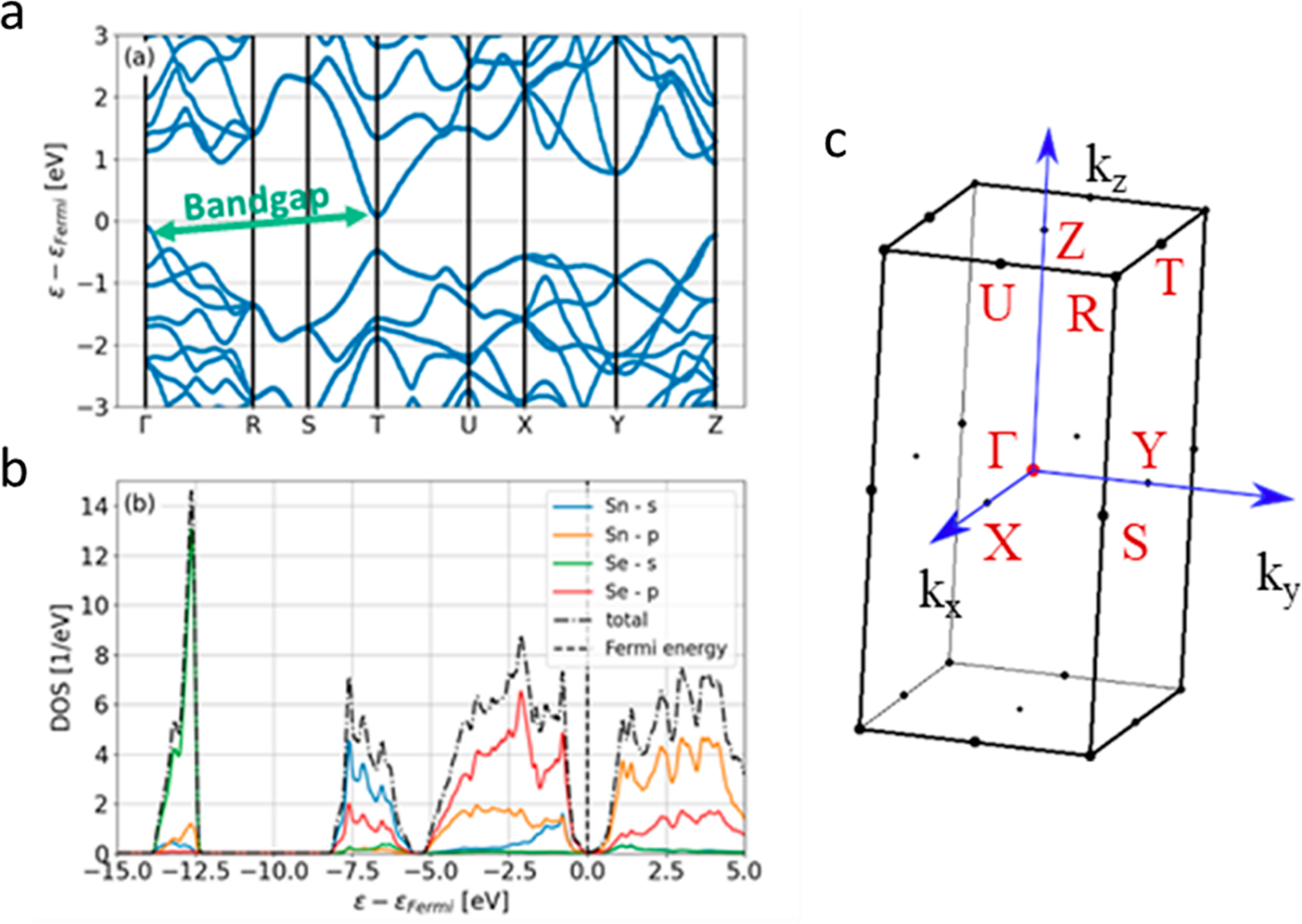
DFT calculation of γ-SnSe: (a) band structure, (b) total and projected DOS, and (c) the orthorhombic Brillouin zone.

The ultranarrow band gap is also consistent with the band structure calculated using DFT. The PBE functional employed here was tested in previous studies and found to reasonably describe the band gaps in the IV–VI monochalcogenides.^51^ The calculated band structure and DOS of γ-SnSe are shown in Figure 4. The band gap is indirect, with a value of 0.1 eV, and the smallest direct transition occurs at the T point with a value of 0.55 eV, as one can see in Figure 4a. For further validation of the value of the band gap, and as an estimation of the uncertainty, we repeated the calculation with the HSE exchange-correlation functional.^48,49^ Within this calculation, we obtain a band gap of 0.17 eV, which is still narrow but larger than the PBE value. We note that the value of the band gap is within the typical uncertainty of DFT calculations. However, it still indicates a very narrow band gap. These results correlate with the absorbance measurements (Figure 3b) and validate the different band gap of γ-SnSe from that of α-SnSe, which is known to possess a much larger indirect band gap.^33,39,40^

Furthermore, this phase is a layered structure in which the layers are in the *y*–*z* plane as calculated in this work. The maximum of the valence band in the γ phase is at the Γ point, and the minimum of the conduction band is at the T point (Figure 4). Thus, electrons in the conduction band will move within the layers, as the T point is in the k_*y*_–k_*z*_ plane.

It is clear from Figure 4b that the projected DOS of the s orbital of the Sn atoms is close to the Fermi energy, which is the lone pair. From this projected DOS, we can determine that the p orbitals of both selenium and tin atoms are the dominant orbitals involved in the chemical bonds.

**Figure 5:**
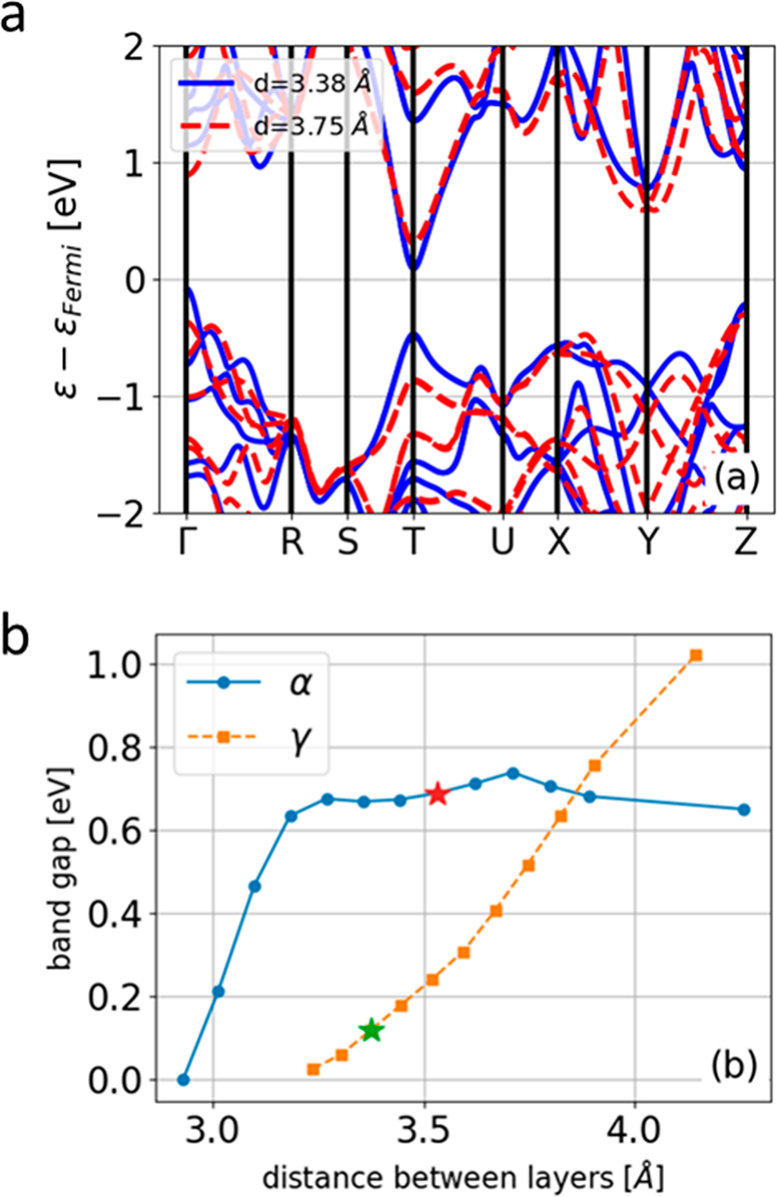
Band structure and energy gap as a function of the distance between layers, *d*. (a) Band structure of γ-SnSe with the equilibrium layer distance and with larger distance as indicated in the legend and (b) band gap in γ-SnSe and α-SnSe as a function of *d*. The stars represent the equilibrium interlayer distances.

To gain better insight into the origin of the narrow band gap of the γ phase, we hypothesized that it is driven by the shorter intralayer distance in the γ phase relative to that in α-SnSe, 3.37 Å versus 3.53 Å. To test this hypothesis, we calculated the band structure in both phases and evaluated the band gap at several interlayer separations, as shown in Figure 5. In the γ phase, the band gap changes monotonically with interlayer separation as expected. In contrast, in the α phase the effect of this distance on the band gap is less significant and furthermore is not monotonic due to a more complex behavior of the band structure. Thus, the interlayer separation is only one of the factors determining the narrow gap in the γ phase, together with the structure of the layers, which is not the same in α- and γ-SnSe.

**Figure 6:**
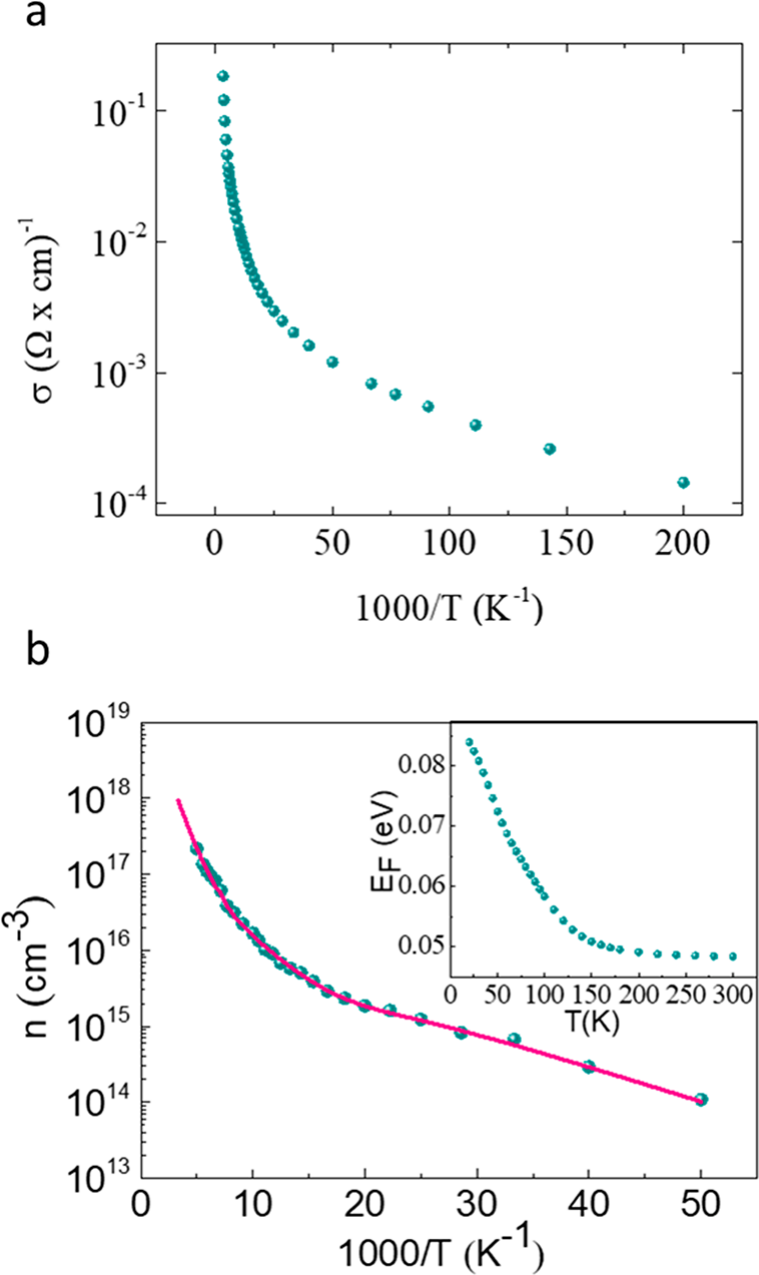
(a) Electrical conductivity as a function of 1000/*T*. Note the high conductivity even at 5 K. (b) Carrier concentration as determined from Hall measurements. The solid line is the fit to the results using the Fermi energies shown in the inset.

Figure 6a shows a plot of conductivity (σ) as a function of inverse temperature. The room-temperature conductivity is ∼0.2 (Ω cm)^−1^. Furthermore, even at 5 K the conductivity is as high as 1.45 × 10^–4^ (Ω cm)^−1^. To better understand this behavior, we performed Hall measurements using a Hall bar configuration in the temperature range of 20–300 K. To eliminate the offset voltage due to misalignment between the Hall contacts, we measured the Hall voltage in opposite directions of the magnetic field at each temperature and subtracted the results, i.e., . We note, however, that the carrier concentration can be extracted from these measurements only for temperatures of ≤200 K. At higher temperatures, the carrier concentration is dominated by intrinsic excitation, i.e., *n* ≈ *p*. In this case , where *V*_H_ is the measured Hall voltage, *w* is the distance between the Hall contacts, *B* is the magnitude of the applied magnetic field, *V* is the potential difference between the source and the drain, and *L* is the distance between them. With these notations,  is the Hall field, *E*_H_, and  is the electric field in the direction of the current. Figure 6b shows the temperature dependence of the majority carrier density, i.e., electrons in this case, as was deduced from the Hall measurements. Apparently, even at 25 K, the electron density is ∼10^14^ cm^–3^ and as high as 2 × 10^17^ cm^–3^ at 200 K. The solid line in the figure is the result of our fitting procedure described below. Note that at higher temperatures the carrier concentration is close to the intrinsic concentration, due to the very small energy gap, with a density of ∼8 × 10^17^ cm^–3^ at room temperature.

Due to the ultranarrow band gap of the system, to evaluate the electron concentration as a function of temperature, we need to use here Fermi–Dirac statistics rather than the more conventional Boltzmann statistics. In this case, the free electron (*n*) and hole (*p*) concentrations are given by^52^
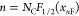
1
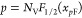
2where , ,  is the Fermi–Dirac integral on the order of ^1^/_2_, and , *E*_C_ and *E*_V_ represent the bottom of the conduction band and the top of the valence band, respectively, *E*_F_ is the Fermi energy, *m*_e_ (*m*_h_) is the (density of states) effective mass of the of electrons (hole), *k* is the Boltzmann constant, and *T* is the temperature. The energetic position of the Fermi energy is determined by the charge neutrality condition.

The temperature dependence of electron concentration, shown in Figure 6b, suggests the presence of two donor-like states. Therefore, the charge neutrality condition used here is
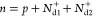
3where  (*i* = 1 or 2) is the concentration of ionized donors from levels *E*_d1_ and *E*_d2_. To evaluate the energetic position and density of these donor levels and the position of the Fermi energy as a function of temperature, we used a self-consistent fitting procedure. We first estimated the energetic position and densities of the donor states from the Hall results, inserted these estimations into the charge neutrality equation, solved numerically for *E*_F_, and used eq 1 to fit the calculated electron concentration to the experimental results. We then refined the values used for the donor levels until the best fit was obtained. This procedure yields a very shallow donor-like level (*E*_d1_), positioned 17 ± 2 meV below the conduction band with an *N*_d1_ of ≈2 × 10^15^ cm^–3^ and a deeper level (*E*_d2_) at 44 ± 3 meV below the conduction band with an *N*_d2_ of ≈6 × 10^17^ cm^–3^. The position of the Fermi energy as a function of temperature is shown in the inset of Figure 6b. The carrier concentration calculated by this procedure is presented by the solid line in Figure 6b and shows a good fit to the experimental data along the entire temperature range. Note that the calculated carrier concentration extends up to 300 K.

**Figure 7:**
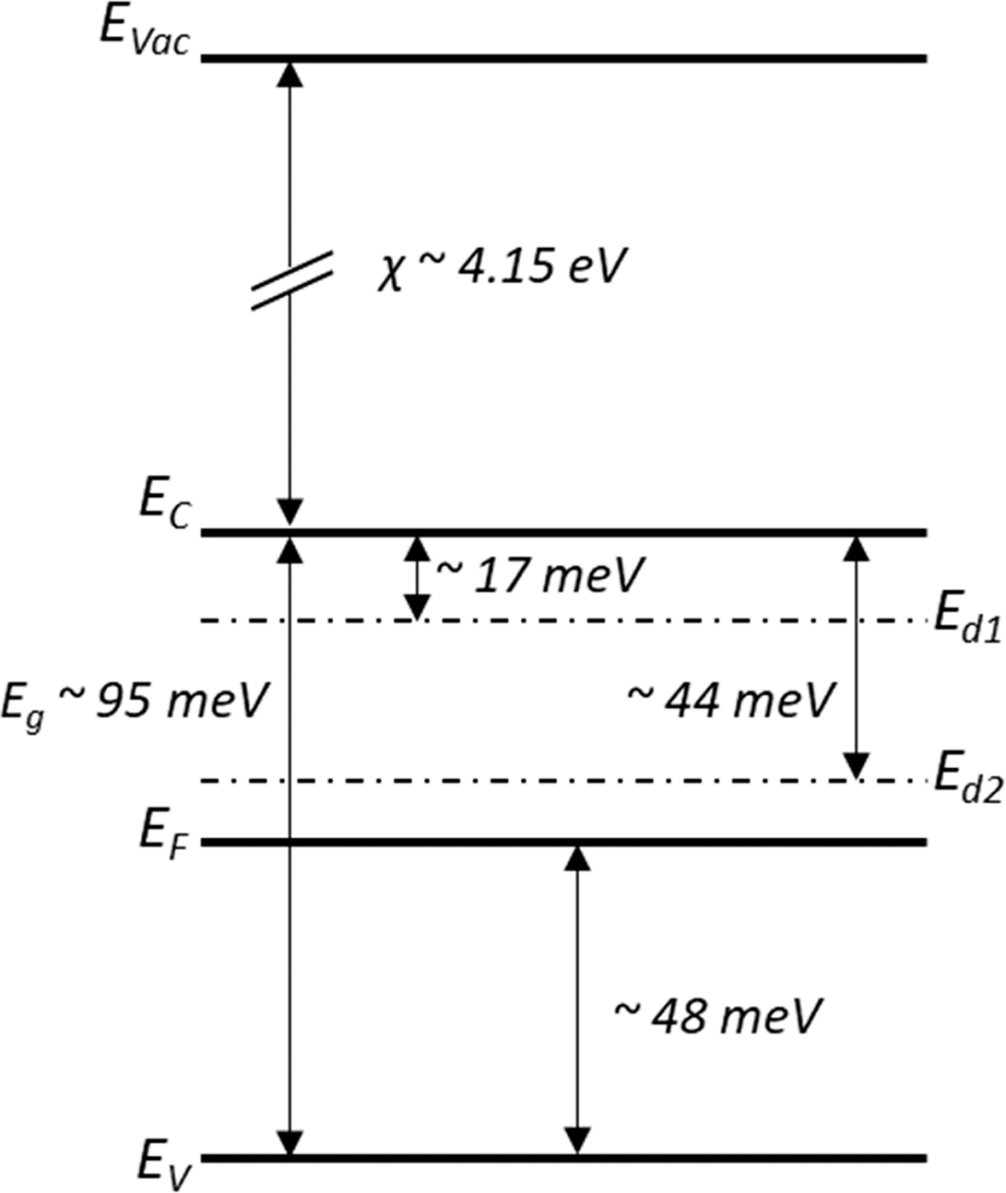
Band diagram at room temperature of γ-SnSe.

The room-temperature work function of the samples was evaluated by kelvin probe measurements (KP Technologies) using a gold electrode, which showed a (surface) work function of 4.2 ± 0.2 eV. According to these results, we can estimate the affinity of this phase to be ∼4.15 ± 0.2 eV at room temperature. In view of all of the results presented above, we can now construct the corresponding band diagram of γ-SnSe at room temperature, as shown in Figure 7.

**Figure 8:**
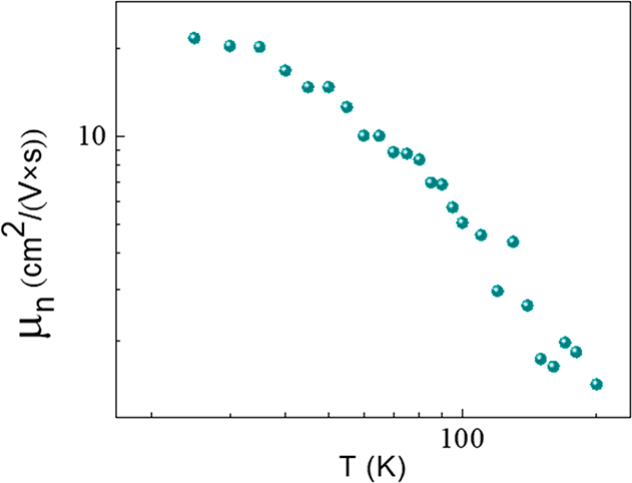
Hall mobility as a function of temperature.

The temperature dependence of the Hall mobility (≤200 K) is presented in Figure 8. At low temperatures, the mobility is approximately constant, probably due to the dominant influence of scattering by (neutral) structural defects. At the higher temperature range (>50 K), it can be described using the power law *μ*_n_ ∝ *T*^–α^ with an α of 1.8 ± 0.1. As discussed above, above 200 K, we can only estimate the difference between the electron and hole mobilities, which is very small, i.e., *μ*_e_ ≈ *μ*_h_, and can be estimated from the electrical conductivity to be ∼0.8 ± 0.2 cm^2^ V^–1^ s^–1^.

The ultranarrow band gap and the high electrical conductivity found in this study make γ-SnSe a potential nontoxic, earth-abundant material for use in a variety of applications. Its ultranarrow band gap makes it one of the narrowest band gap semiconductors, along with InSb,^53^ Ag_2_Se,^54^ Ti_2_O_3_,^55^ and some of the Hg_1–*x*_Cd_*x*_Te compounds^56^ (depending on *x*). Its potential applications extend from infrared detectors, thermal imaging to photothermal conversion, and thermoelectric devices.^43,55,57^ The absorption onset at ∼12.9 μm makes it suitable for thermal imaging and photodetection in the 8–12 μm atmospheric window as well as in the 3–5 μm range. Additionally, its ultranarrow band gap will create a type I band alignment with most semiconductors, which can also be utilized in heterojunction applications such as suggested for PbSe.^58^ For example, the good lattice match and chemical compatibility with the α-SnSe phase are likely to facilitate the formation of a high-quality heterojunction with low concentration of interface defects. The γ-SnSe phase can also be utilized to form an ohmic contact between the absorber and the back contact metal in α-SnSe-based solar cells, which are very sensitive to the formation of a Schottky barrier there.^59^ The new chemical and physical properties along with these potential applications make this phase an exciting new material that will be subject to further studies.

## Conclusions

In this work, we studied the electronic properties of the recently discovered γ-SnSe phase. Unexpectedly, we found a significant different narrow band gap of ∼95 meV compared to α-SnSe with band gap of ∼1 eV. These results were further supported by DFT calculations. Consequently, the carrier concentration at temperatures above 200 K, and thus the electrical conductivity, are dominated by intrinsic excitations. Our Hall measurements indicate the presence of two donor levels positioned at ∼17 and ∼44 meV below the conduction band. These levels are responsible for the high carrier concentration found at low temperatures and for the relatively high conductivity even at 5 K. Hall mobility results show that the temperature dependence of the electron mobility at temperatures between 50 and 200 K can be described by the power law *μ*_n_ ∝ *T*^–1.8^. The results also indicate that, at least for the range of 200–300 K, electron and hole mobilities are quite similar. The newly discovered electrical properties of γ-SnSe thin films open a variety of new application options for this newly discovered binary phase semiconductor material.

## Supporting Information Available

Additional characterization data, including HR-SEM and AFM data, optical absorption data and an illustration of the Hall configuration (PDF)
