# Supplementary material for: Electrical and Optical Properties of γ-SnSe: A New Ultra-narrow Band Gap Material
Source: ACS Appl Mater Interfaces. 2023 Mar 15;15(12):15668–75. doi: 10.1021/acsami.2c22134 (PMC10064319; doi:10.1021/acsami.2c22134)
Supplement: Supplementary file 1 — am2c22134_si_001.pdf [file am2c22134_si_001.pdf]

Supporting Information

Electrical and Optical Properties of γ-SnSe – A New Ultra-narrow Band Gap Material

Noy Zakay[a,b], Adi Schlesinger[c], Uri Argaman[a], Long Nguyen[a], Nitzan Maman[b], Bar Koren[a,b], Meital Ozeri[d], Guy Makov[a], Yuval Golan*[a,b], and Doron Azulay*[c,d]*

(a) Department of Materials Engineering, Ben-Gurion University of the Negev, Beer-Sheva 8410501, Israel

(b) Ilse Katz Institute for Nanoscale Science and Technology, Ben-Gurion University of the Negev, Beer-Sheva 8410501, Israel

(c) Azrieli, Jerusalem College of Engineering, Jerusalem 9103501, Israel

(d) Racah Institute of Physics, The Hebrew University, Jerusalem 9190401, Israel

*azulay.doron@mail.huji.ac.il

*ygolan@bgu.ac.il

*makovg@bgu.ac.il

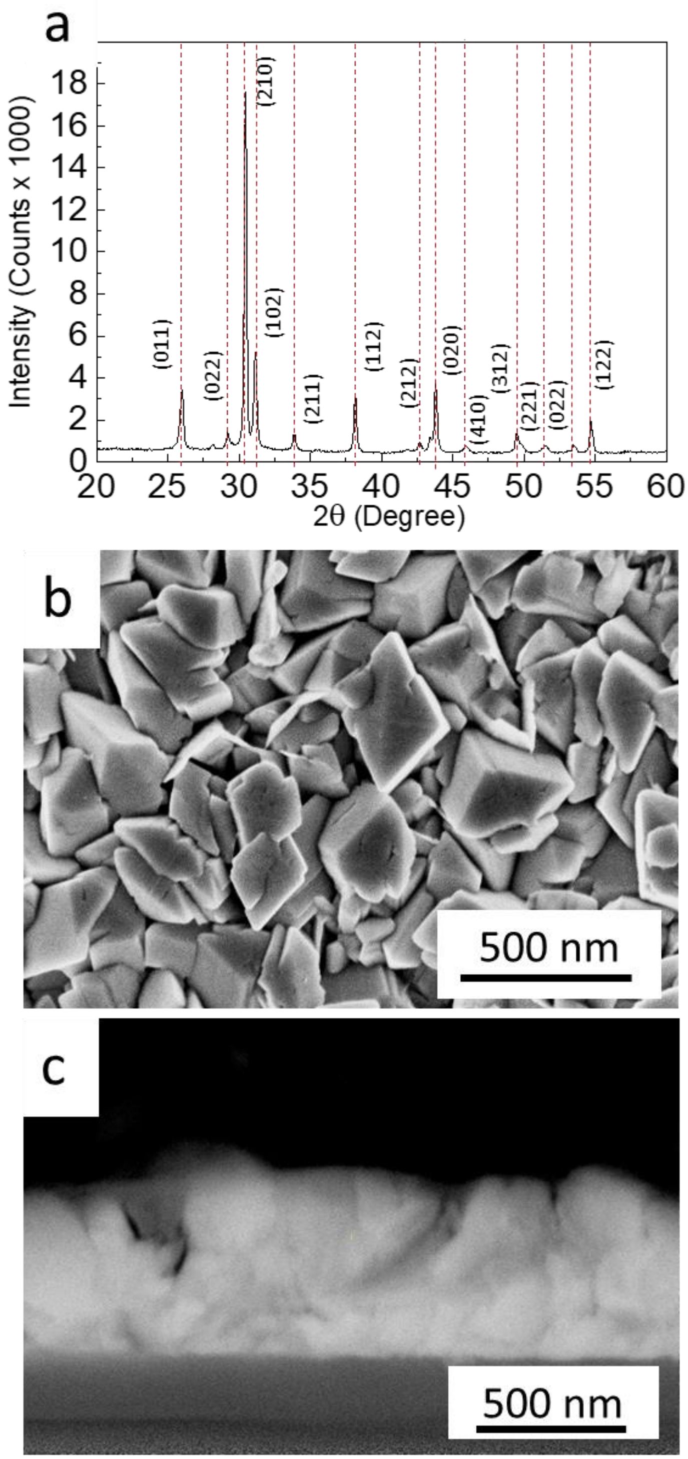

Figure S1. Characterization of γ-SnSe thin films deposited onto GaAs (100) (a) XRD, (b) Plan-view HR-SEM image, and (c) Cross-section HR-SEM image.

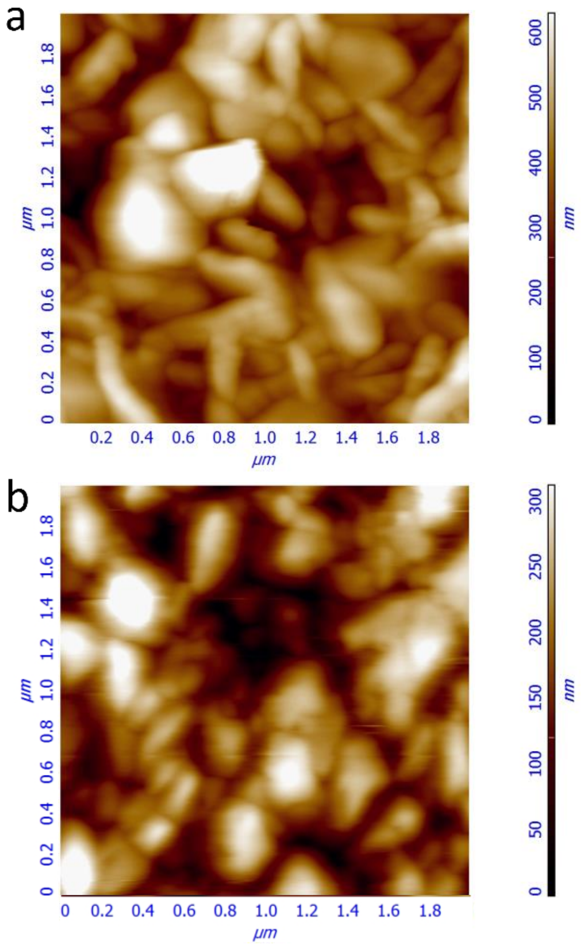

Figure S2. AFM images of γ-SnSe thin films deposited onto (a) Quartz and (b) GaAs substrates.

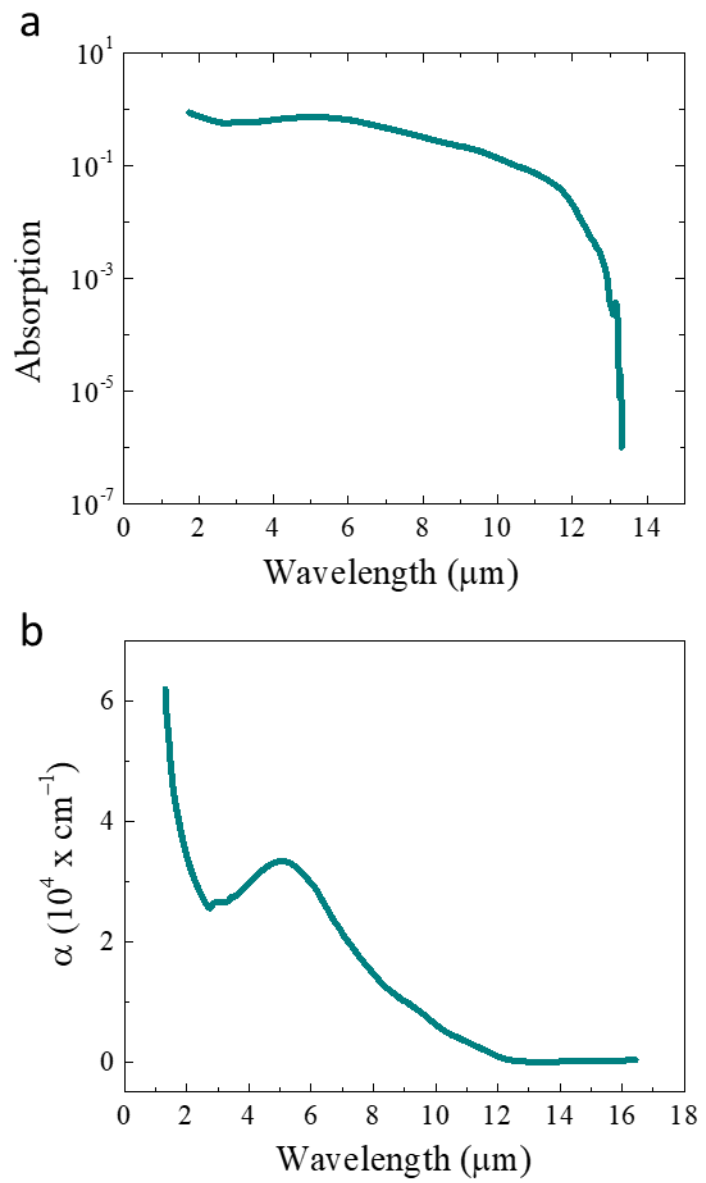

Figure S3. Optical measurements of γ- SnSe thin films deposited onto GaAs substrate (a) Absorption spectrum with *y*-axis in logarithmic scale (b) Absorption coefficient spectrum.

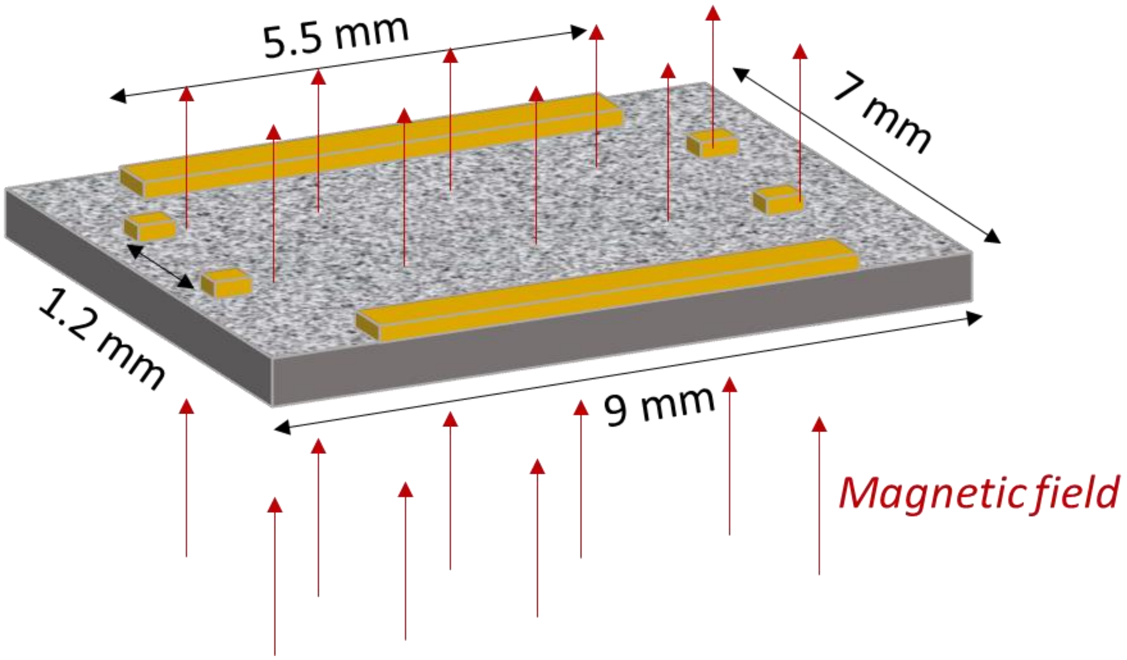

Figure S4. A schematic illustration of the Hall measurements setup, showing Au contacts on the γ-SnSe thin film.
